# Why Most Published Research Findings Are False: Problems in the Analysis

**DOI:** 10.1371/journal.pmed.0040168

**Published:** 2007-04-24

**Authors:** Steven Goodman, Sander Greenland

The article published in *PLoS Medicine* by Ioannidis [1] makes the dramatic claim in the title that “most published research claims are false,” and has received extensive attention as a result. The article does provide a useful reminder that the probability of hypotheses depends on much more than just the *p*-value, a point that has been made in the medical literature for at least four decades, and in the statistical literature for decades previous. This topic has renewed importance with the advent of the massive multiple testing often seen in genomics studies.

Unfortunately, while we agree that there are more false claims than many would suspect—based both on poor study design, misinterpretation of *p*-values, and perhaps analytic manipulation—the mathematical argument in the *PLoS Medicine* paper underlying the “proof” of the title's claim has a degree of circularity. As we show in detail in a separately published paper [2], Dr. Ioannidis utilizes a mathematical model that severely diminishes the evidential value of studies—even meta-analyses—such that none can produce more than modest evidence against the null hypothesis, and most are far weaker. This is why, in the offered “proof,” the only study types that achieve a posterior probability of 50% or more (large RCTs [randomized controlled trials] and meta-analysis of RCTs) are those to which a prior probability of 50% or more are assigned. So the model employed cannot be considered a proof that most published claims are untrue, but is rather a claim that no study or combination of studies can ever provide convincing evidence.

The two assumptions that produce the above effect are:

Calculating the evidential effect only of verdicts of “significance,” i.e., *p* ≤ 0.05, instead of the actual p-value observed in a study, e.g., *p* = 0.001.
Introducing a new “bias” term into the Bayesian calculations, which even at a described “minimal” level (of 10%) has the effect of very dramatically diminishing a study's evidential impact.

In addition to the above problems, the paper claims to have proven something it describes as paradoxical; that the “hotter” an area is (i.e., the more studies published), the more likely studies in that area are to make false claims. We have shown this claim to be erroneous [2]. The mathematical proof offered for this in the *PLoS Medicine* paper shows merely that the more studies published on any subject, the higher the absolute number of false positive (and false negative) studies. It does not show what the papers' graphs and text claim, viz, that the number of false claims will be a higher proportion of the total number of studies published (i.e., that the positive predictive value of each study decreases with increasing number of studies).

The paper offers useful guidance in a number of areas, calling attention to the importance of avoiding all forms of bias, of obtaining more empirical research on the prevalence of various forms of bias, and on the determinants of prior odds of hypotheses. But the claims that the model employed in this paper constitutes a “proof” that most published medical research claims are false, and that research in “hot” areas is most likely to be false, are unfounded.
